# Experimental quantification of frame‐level active window duration and duty cycle in high frame rate x‐ray detectors

**DOI:** 10.1002/mp.70627

**Published:** 2026-08-12

**Authors:** Ruiran Lai, Linying Zhan, Bujar Mehmeti, Ran Zhang, Ke Li, Guang‐Hong Chen

**Affiliations:** ^1^ Department of Medical Physics University of Wisconsin‐Madison Madison Wisconsin USA; ^2^ Department of Radiology University of Wisconsin‐Madison Madison Wisconsin USA; ^3^ Department of Imaging Physics University of Texas MD Anderson Cancer Center Houston Texas USA; ^4^ Department of Interventional Radiology University of Texas MD Anderson Cancer Center Houston Texas USA

**Keywords:** photon counting detector, temporal resolution, x‐ray imaging

## Abstract

**Background:**

Detector frame rate is commonly used as the primary metric to characterize the temporal behavior of x‐ray detectors in medical imaging systems. However, frame rate alone specifies only the sampling rate of image frames and does not determine how x‐ray interaction signals are temporally integrated to form the measured signal within each frame. In medical imaging applications, an important frame‐level component of detector temporal performance is characterized by the actual x‐ray active window and by the fraction of the frame time during which the detector is actively exposed to x‐rays. These frame‐level timing characteristics are crucial for assessing detector radiation dose efficiency, yet they are rarely measured experimentally.

**Purpose:**

To develop and experimentally validate a method for quantifying the frame‐level temporal behavior of high frame rate x‐ray detectors beyond frame rate alone, by directly measuring the actual x‐ray active window duration and the detector temporal duty cycle during active x‐ray exposure.

**Methods:**

A rotating test object with sharp lead‐foil edges was imaged using a high frame rate photon‐counting detector over a wide range of nominal frame‐rate settings up to 4500 fps. An independent laser tachometer was used to measure the angular velocity of the rotating object. Motion blur was analyzed using simple kinematic models that establish a direct relationship between the detector active window duration and the angular width of the blurred edge‐spread function (ESF) in projection images. The active window duration was estimated from the measured angular blur width. The experimentally measured frame rate was determined independently from the angular displacement of recurring features between frames. The detector duty cycle was computed as the ratio of the measured active window duration to the measured frame time.

**Results:**

The experimentally measured frame rate closely matched the nominal setting over the full tested range, with a linear relationship characterized by a slope of 1.002 and $R^{2}=0.9999$. In contrast, the actual x‐ray active window duration was consistently shorter than the frame time and decreased with increasing frame rate. A linear dependence of active window duration on frame time indicated a frame‐rate‐independent inactive window of approximately 0.15 ms per frame. As a result, the detector temporal duty cycle decreased systematically from above 90% at low frame rates to below 40% at the highest tested settings.

**Conclusions:**

Nominal frame rate alone is insufficient to characterize detector temporal performance, because reduced active window duration within each frame substantially lowers the detector temporal duty cycle at high frame rates, with direct implications for radiation dose utilization and efficiency.

## 1 INTRODUCTION

High‐frame‐rate x‐ray detectors are important for computed tomography (CT) data acquisition and time‐resolved x‐ray imaging applications.^1^, ^2^, ^3^, ^4^, ^5^, ^6^ These detectors may operate using different signal‐formation mechanisms, including conventional energy‐integrating detection (EID) and photon‐counting detection (PCD). In all cases, however, the measured projection data are formed through finite temporal integration within each frame. Therefore, characterizing the temporal response of high‐frame‐rate x‐ray detectors is important for understanding their performance limits and for tailoring their use in specific imaging applications.

Increasing clinical and technical demands have also placed growing emphasis on acquisition throughput and robustness to motion in in vivo imaging. The need to mitigate inadvertent patient motion, as well as unavoidable cardiac and respiratory motion, has driven x‐ray imaging systems toward progressively shorter acquisition times. In CT, this trend has resulted in ever shorter gantry rotation times, requiring acquisition of a complete dataset within a fraction of a second. As a direct consequence, modern CT and dynamic x‐ray imaging systems require detector readout at frame rates on the order of 10,000 frames per second to meet throughput and motion‐mitigation requirements.

Temporal performance is therefore a critical factor in x‐ray and CT imaging, and substantial effort has been devoted to characterizing temporal resolution in CT. CT temporal resolution is commonly defined as the effective time span over which projection data contribute to a reconstructed image. Established frameworks, such as the temporal sensitivity profile, quantify image‐level temporal resolution by describing the temporal weighting of projection data and defining resolution through metrics such as the full width at half maximum.^7^ Experimental implementations have employed impulse‐response techniques^8^, ^9^ and motion phantoms with task‐based image quality analyses, including temporal modulation transfer function–based methods.^10^, ^11^, ^12^ Together, these studies have improved understanding of image‐level and system‐level temporal resolution in CT.

However, CT temporal resolution metrics characterize how projection data are combined during reconstruction and do not directly describe how x‐rays are temporally sampled at the detector prior to reconstruction. As CT systems are pushed toward increasingly short acquisition times and extremely high detector frame rates, detector‐level temporal behavior becomes a distinct and increasingly important consideration. In this regime, the duration of active window within each frame and the fraction of the frame time effectively used for accumulating detector signal can substantially influence motion blur and radiation dose efficiency, even when the nominal frame rate is fixed.

Detector frame rate alone therefore does not fully characterize detector temporal behavior. Frame rate specifies the sampling rate of image frames, but it does not determine the active window duration within each frame or the detector duty cycle, nor does it describe other physical mechanisms contributing to detector lag. These frame‐level timing characteristics directly affect dose efficiency, particularly at high frame rates where inactive windows can constitute a substantial fraction of each frame period. Yet the actual x‐ray active window duration and temporal duty cycle are rarely reported and are often implicitly assumed to match nominal frame timing specified by the manufacturer.

At the detector level, temporal response has also been studied through related concepts such as detector lag, lag correction, temporal aperture, and temporal modulation transfer function (MTF). Prior work on flat‐panel x‐ray energy integrating detectors has modeled residual signal carryover from previous frames and developed lag‐correction methods, and has further examined the influence of detector lag on CT image noise properties.^13^, ^14^, ^15^, ^16^, ^17^ Moving‐edge methods have also been used to measure the temporal MTF of fluoroscopic systems, where the measured temporal response can reflect both finite temporal integration and delayed signal components.^18^, ^19^ In scintillator‐based energy‐integrating detectors, such lag effects are commonly associated with delayed scintillation‐light emission or other history‐dependent signal carryover between frames. Photon‐counting detectors can also exhibit temporal‐response effects related to charge transport, pulse formation, and signal processing; for example, incomplete charge collection or pulse pileup can affect pulse amplitude, energy‐bin assignment, and count‐rate response.^20^ These effects are conceptually related to detector temporal response, but they are distinct from the frame‐level inactive‐window effect investigated in the present work. Here, we focus on a specific frame‐level timing quantity: the effective x‐ray active‐window duration within each detector frame and the corresponding temporal duty cycle. The proposed method estimates this quantity directly from motion‐induced ESF broadening in projection images, rather than measuring the full temporal MTF or independently modeling history‐dependent lag response.

To avoid conceptual ambiguity, the frame‐level temporal quantities considered in this work are defined explicitly. The frame time refers to the reciprocal of the frame rate. Within each frame time, signal is accumulated only in an active time window, referred to here as the active window duration τ. The remaining portion of the frame time is not recording x‐rays even if they interact with the detector sensor. The temporal duty cycle of detector is defined as τ/T. In the data analysis, the observed frame rate $f_{\mathrm{obs}}$ is used in place of frame rate f when estimating these quantities from experimental measurements.

These frame‐level timing characteristics should not be confused with event‐level detector timing effects, most notably detector dead time. For photon‐counting detectors, detector dead time, typically on nanosecond time scales, describes the recovery time of the pulse processing electronics following individual photon detection events and governs count‐rate‐dependent pulse losses. In contrast, the frame time and the frame‐level active window duration considered here are on the order of milliseconds and are governed by frame‐based readout and acquisition control. This separation of time scales indicates that the quantities measured in this study characterize frame‐level temporal availability rather than event‐level pulse processing.

The purpose of this study is to develop a method for quantifying frame‐level temporal behavior in high‐frame‐rate x‐ray detectors and to experimentally validate this method using a high‐frame‐rate PCD. Using a rotating object with well‐defined geometry, we extracted the actual active window duration from motion‐induced blur in projection images and independently measured the actual frame rate from frame‐to‐frame angular displacements of recurring features. The temporal duty cycle was then computed from the measured active window duration and frame time. This approach provides a practical, reconstruction‐independent means to quantify detector temporal behavior beyond nominal frame rate specifications and to assess its implications for radiation dose efficiency in high‐speed x‐ray imaging systems.

## 2 THEORETICAL MODEL OF MOTION BLUR ARISING FROM TEMPORAL INTEGRATION

When x‐rays interact with an image object, the transmitted photons are converted by the detector into electrical signals, which are subsequently digitized. Regardless of detector operation modes, either EIDs or PCDs, the measured signal arises from two fundamental integration processes: spatial integration over the detector aperture and temporal integration over a finite time window.

For a static object, these spatial and temporal integrations are decoupled: the spatial integration determines spatial resolution, while the temporal integration affects only the signal‐to‐noise ratio. In contrast, when the object is in motion, its configuration changes during the active window. As a result, the measured signal represents a temporal average of the moving object, intrinsically coupling spatial and temporal effects and introducing motion‐induced blur.

Consequently, the temporal performance of a detector governs how faithfully object motion can be captured. A quantitative assessment of detector temporal performance therefore requires explicit modeling of the signal formation process in the presence of object motion. This section presents such a model to establish the foundation for our experimental measurement methods.

For simplicity, the presented model treats the projected object shadow as having ideally sharp boundaries and assumes that the measured ESF broadening is dominated by temporal integration during the detector active window. Other potential sources of image blur, including finite focal‐spot blur and detector‐aperture blur, are not explicitly included in the theoretical derivation.

### 2.1 Theoretical model of motion blur: Translational motion case

We first consider a simplified translational geometry in which an object of width a is placed in direct contact with the detector. Under this configuration, the object width and the motion‐induced blur length can be written without introducing an additional geometric magnification factor. This direct‐contact assumption is therefore used only to simplify the notation in the translational derivation and is not an assumption used in the rotational experimental measurement described below.

**FIGURE 1:**
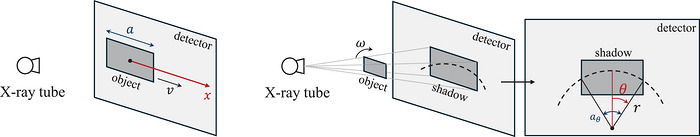
Schematic illustration of the coordinate systems used in the translational and rotational motion‐blur models. In the translational case (left), the object of width a translates along the detector‐plane coordinate x with velocity v. In the rotational case (right), the object rotates with angular velocity ω, and the planar detector image is reparametrized along a circular analysis arc of radius r centered at the projected rotation axis. The angular coordinate θ and the angular object width $a_{\theta }$ are used to describe the shadow profile in the rotational formulation.

As illustrated in Figure 1, the object is assumed to translate at a constant velocity v along the $+\hat{x}$ direction under temporally stable x‐ray illumination. The detector count rate per detector element under open‐beam conditions is denoted by $R_{\mathrm{open}}$. Here, R is used as a generic time‐dependent detector count rate: for a photon‐counting detector, it represents the detected photon count rate, whereas for an energy‐integrating detector, it may be interpreted as the detector output signal, integrated charge, or signal intensity per unit time. When a detector element is shadowed by the object, the corresponding attenuated count rate is denoted by $R_{\mathrm{block}}$. Throughout the analysis, both $R_{\mathrm{open}}$ and $R_{\mathrm{block}}$ are assumed to be time independent.

Under these assumptions, the instantaneous detector count rate at detector position x and time t is modeled as

(1) $$R_{\mathrm{inst}}\left(x;t\right)=\left\{\begin{array}{cc} R_{\mathrm{block}}, & |x-x_{0}\left(t\right)|\leq a/2,\\ R_{\mathrm{open}}, & \text{otherwise}, \end{array}\right.$$

where $x_{0}\left(t\right)$ denotes the instantaneous center position of the translating object. For a stationary object with sharp boundaries, the instantaneous count‐rate distribution is therefore time invariant and exhibits a rectangular spatial profile determined solely by the object width a.

To account for object motion during x‐ray exposure, we assume rigid translation such that spatial coordinates evolve according to x↦x−vt. The measured image intensity profile, defined as the detector signal temporally integrated over the active window $\left[t_{0},t_{0}+\tau \right]$, is then given by

(2) $$I\left(x\right)=\int_{t_{0}}^{t_{0}+\tau }R_{\mathrm{inst}}\left(x-vt\right)\;dt,$$

where τ denotes the active window duration during which the detector signal is actively recorded. Introducing the change of variables u=x−vt yields

(3) $$I\left(x\right)=\frac{1}{v}\int_{x-v(t_{0}+\tau )}^{x-vt_{0}}R_{\mathrm{inst}}\left(u\right)\;du.$$

To make the connection between motion and spatial blurring explicit, we define the rectangular kernel

(4) $$\Pi_{\Delta x}\left(s\right)=\left\{\begin{array}{cc} 1, & 0\leq s\leq \Delta x,\\ 0, & \text{otherwise}, \end{array}\right.\;\Delta x=v\tau ,$$

which represents the spatial distance traveled by the object during the active window. Using the standard definition of convolution, one obtains

(5) $$\left(R_{\mathrm{inst}}*\Pi_{\Delta x}\right)\left(x\right)=\int_{x-\Delta x}^{x}R_{\mathrm{inst}}\left(u\right)\;du.$$

Comparing Equations (3) and (5), the measured profile can be written as

(6) $$I\left(x\right)=\frac{1}{v}\left(R_{\mathrm{inst}}*\Pi_{\Delta x}\right)\left(x-vt_{0}\right),$$

the active window start time $t_{0}$ introduces only a spatial shift and does not affect the blurring kernel width Δx as shown in Equation (4). Without loss of generality, we therefore set $t_{0}=0$, yielding

(7) $$I\left(x\right)=\frac{1}{v}\left(R_{\mathrm{inst}}*\Pi_{\Delta x}\right)\left(x\right).$$

Equation (7) shows that motion blur arises from convolution of the instantaneous count‐rate profile with a rectangular kernel whose width is determined by the distance traveled during the active window.

Using the definition of $R_{\mathrm{inst}}$ in Equation (1) and setting $x_{0}\left(t_{0}\right)=0$, the integral in Equation (7) can be evaluated by introducing the overlap length

(8) $$L\left(x\right)=\mathrm{length}\left([x-\Delta x,x]\cap [-a/2,a/2]\right).$$

The measured profile can then be written as

(9) $$\begin{array}{cc} I(x) & =\frac{1}{v}\left[R_{\mathrm{block}}L\left(x\right)+R_{\mathrm{open}}\left(\Delta x-L(x)\right)\right]\\ & =R_{\mathrm{open}}\tau +\frac{L(x)}{\Delta x}\left(R_{\mathrm{block}}\tau -R_{\mathrm{open}}\tau \right). \end{array}$$

Defining

(10) $$I_{\mathrm{open}}=R_{\mathrm{open}}\tau ,\;I_{\mathrm{block}}=R_{\mathrm{block}}\tau ,$$

and introducing the normalized image contrast

(11) $$\eta \left(x\right)=\frac{I_{\mathrm{open}}-I\left(x\right)}{I_{\mathrm{open}}-I_{\mathrm{block}}},$$

it follows directly from Equation (9) that

(12) $$\eta \left(x\right)=\frac{L(x)}{\Delta x},$$

with 0≤η(x)≤1. As detailed in the Appendix, the normalized contrast profile admits a unified explicit representation that is valid across both a>Δx and a≤Δx cases. To this end, we introduce the auxiliary quantities

(13) $$w\equiv \min\left(a,\Delta x\right),\;\eta_{\max}\equiv \min\;\left(1,\frac{a}{\Delta x}\right),$$

which respectively characterize the effective transition width and the maximum achievable normalized contrast. With these definitions, the normalized contrast profile can be written in a regime‐agnostic piecewise form as

(14) $$\eta \left(x\right)=\left\{\begin{array}{cc} 0, & x\leq -a/2,\\ \frac{x+a/2}{\Delta x}, & -a/2<x<-a/2+w,\\ \eta_{\max}, & -a/2+w\leq x\leq a/2+\Delta x-w,\\ \frac{a/2+\Delta x-x}{\Delta x}, & a/2+\Delta x-w<x<a/2+\Delta x,\\ 0, & x\geq a/2+\Delta x. \end{array}\right.$$

**FIGURE 2:**
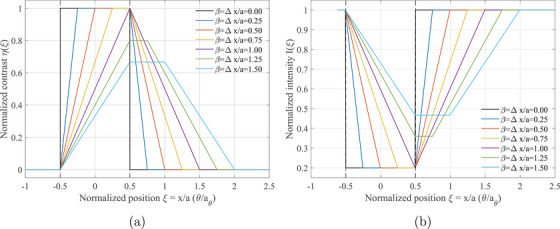
Effect of motion blur on normalized contrast and intensity profiles. (a) Normalized image contrast profiles η(ξ) for a moving object with sharp edges, plotted for different values of the normalized blur parameter β=Δx/a=vτ/a. The normalized detector coordinate is ξ=x/a, with object boundaries at ξ=±0.5. (b) Corresponding normalized intensity profiles I(ξ) extracted along the dashed detector line in the simulated images, with $I_{\mathrm{block}}/I_{\mathrm{open}}=0.2$.

As illustrated in Figure 2, the normalized contrast profile and the corresponding raw image intensity profile exhibits a trapezoidal form whose detailed structure depends on the ratio between the object width a and the blurring kernel width Δx=vτ. For slow object motion (a>Δx, i.e., v<a/τ), the active window duration can be fully contained within the object shadow for a range of detector positions, resulting in a unit‐height contrast plateau bounded by two linear transition regions whose spatial extent scales with Δx. In contrast, for fast object motion (a≤Δx, i.e., v≥a/τ), full shadowing is no longer possible during the active window; the transition regions become object‐limited with fixed width a, while the maximum attainable normalized contrast is reduced to a/Δx=a/(vτ). The boundary case a=Δx corresponds to a triangular contrast profile in which the plateau collapses to a single point and the peak contrast varies continuously between the two regimes.

Beyond providing physical insight into motion‐induced image blur, the above model also enables a direct experimental approach for quantifying the detector active window duration τ from measured contrast profiles. In the regime with a>Δx, the blurring kernel width Δx can be determined directly from the spatial width of the linear transition regions, yielding τ=Δx/v when the object translation speed v is known. In the regime with a≤Δx, the transition widths no longer encode Δx and instead reflect the object size a; in this case, the active window duration can be inferred from the reduced plateau height $\eta_{\max}=a/\Delta x$ or from the width of the plateau Δx−a. Taken together, these complementary observables allow the same blur model to be used to extract τ across both regimes, provided that the appropriate profile feature is used for each.

### 2.2 Theoretical model of motion blur: Rotational motion case

The translational motion–blur analysis presented above extends directly to rigid rotational motion through a reparameterization of the detector coordinate. Specifically, instead of a linear detector coordinate x, we introduce an angular coordinate θ defined along a circular profile of radius r centered at the rotation axis. For an object undergoing rigid rotation at a constant angular velocity ω, the angular position of the object center evolves as $\theta_{0}\left(t\right)=\theta_{0}\left(0\right)+\omega t$. During an active window of duration τ, the object therefore sweeps an angular distance Δθ=ωτ.

Under these conditions, the instantaneous count‐rate distribution at the detector retains the same rectangular form as in the translational case, with the object shadow now defined in the angular domain by an angular width $a_{\theta }$. Because the time dependence of the object position enters only through a uniform shift in θ, the temporal integration over the active window proceeds identically to the translational case after the change of variables x→θ. Consequently, all expressions derived for translational motion carry over directly, with the substitutions Δx→Δθ and v→ω. Although the detector elements lie on a planar array, the rotational formulation does not require a physically curved detector geometry. Instead, the planar detector image is reparametrized by sampling detector pixel values along circular analysis arcs centered at the projected rotation axis. The angular coordinate θ therefore represents a coordinate transformation of detector‐plane image data, rather than the geometry of a curved detector, as illustrated in Figure 1.

Following the unified formulation introduced for translational motion, the two rotational regimes can be described using the auxiliary quantities

(15) $$w_{\theta }\equiv \min\left(a_{\theta },\Delta \theta \right),\;\eta_{\max}\equiv \min\;\left(1,\frac{a_{\theta }}{\Delta \theta }\right),$$

which respectively represent the effective angular transition width and the maximum achievable normalized contrast.

With these definitions, the normalized contrast profile for rotational motion can be written in a regime‐agnostic piecewise form as

(16) $$\eta \left(\theta \right)=\left\{\begin{array}{cc} 0, & \theta \leq -a_{\theta }/2,\\ \frac{\theta +a_{\theta }/2}{\Delta \theta }, & -a_{\theta }/2<\theta <-a_{\theta }/2+w_{\theta },\\ \eta_{\max}, & -a_{\theta }/2+w_{\theta }\leq \theta \leq a_{\theta }/2+\Delta \theta -w_{\theta },\\ \frac{a_{\theta }/2+\Delta \theta -\theta }{\Delta \theta }, & a_{\theta }/2+\Delta \theta -w_{\theta }<\theta <a_{\theta }/2+\Delta \theta ,\\ 0, & \theta \geq a_{\theta }/2+\Delta \theta . \end{array}\right.$$

Equation (16) shows that rotational motion blur is characterized by two linear transition regions and a central plateau, whose detailed structure depends on the relative magnitudes of $a_{\theta }$ and Δθ=ωτ. For slow rotation ($\Delta \theta \leq a_{\theta }$), a fully shadowed angular region exists during the active window, resulting in a unit‐height contrast plateau bounded by transition regions of angular width Δθ. In contrast, for fast rotation ($\Delta \theta >a_{\theta }$), no angular location remains shadowed throughout the active window; the transition regions become object‐limited with fixed angular width $a_{\theta }$, while the peak contrast decreases to $a_{\theta }/\Delta \theta$. The boundary case $\Delta \theta =a_{\theta }$ corresponds to a triangular contrast profile, providing a continuous transition between the two regimes.

Because Δθ is an angular quantity, it is invariant under geometric magnification. When $\Delta \theta \leq a_{\theta }$, the detector active window duration can therefore be determined directly from the measured angular blur width according to

(17) $$\tau =\frac{\Delta \theta }{\omega },$$

independent of the object–detector distance or the rotation radius r. If geometric magnification scales both the projected arc length and radius by the same factor, their ratio remains. This magnification invariance renders the rotational formulation particularly well suited for practical and robust experimental measurement of detector temporal integration behavior.

A key practical limitation of the rotational approach must, however, be recognized. The above formulation assumes that the angular distance swept during a single active window, Δθ=ωτ, is sufficiently smaller than 2π such that the object does not revisit the same angular location within one active window, corresponding to a single‐pass regime. Under this condition, each angular position is shadowed at most once during the active window, and the measured contrast profile is accurately described by the trapezoidal model derived above.

When Δθ becomes comparable to or exceeds 2π, the intrinsic periodicity of rotational motion introduces an additional angular scale beyond the object shadow width $a_{\theta }$. In this wrap‐around regime, the swept angle can be expressed as

$$\Delta \theta =2\pi n+\delta ,\;n=\left\lfloor \frac{\Delta \theta }{2\pi }\right\rfloor ,\;0\leq \delta <2\pi ,$$

where n denotes the number of complete revolutions during a single active window and δ is the residual swept angle. For n≥1, a given angular location may be shadowed multiple times within the same active window, causing the measured image to reflect a rotational time average rather than a single‐pass motion‐blur profile. In the limit of many revolutions, the normalized contrast approaches the angular duty factor $a_{\theta }/\left(2\pi \right)$ and becomes approximately independent of θ.

In this wrap‐around regime, the trapezoidal contrast model underlying the present method no longer applies, and reliable extraction of the detector active window duration from blur geometry becomes impractical. This limitation is intrinsic to rotational motion and arises solely from its periodic nature, with no direct analogue in the translational case.

Despite this constraint, rotational motion remains highly attractive for experimental characterization of detector temporal integration behavior. By virtue of the magnification invariance of the angular blur width Δθ=ωτ, the rotational approach eliminates the need for precise distance measurements required in translational motion experiments. Provided that the rotation speed is chosen such that Δθ≪2π, thereby avoiding angular wrap‐around, rotational motion offers a robust, geometrically insensitive, and experimentally convenient means of measuring detector active window duration.

## 3 EXPERIMENTAL METHODS AND MATERIALS

As discussed in the previous section, owing to the invariance of the angular formulation in Equation (17) with respect to geometric magnification, all experimental measurements in this work were performed using rotational motion.

### 3.1 Experimental setup and data acquisition parameters

**FIGURE 3:**
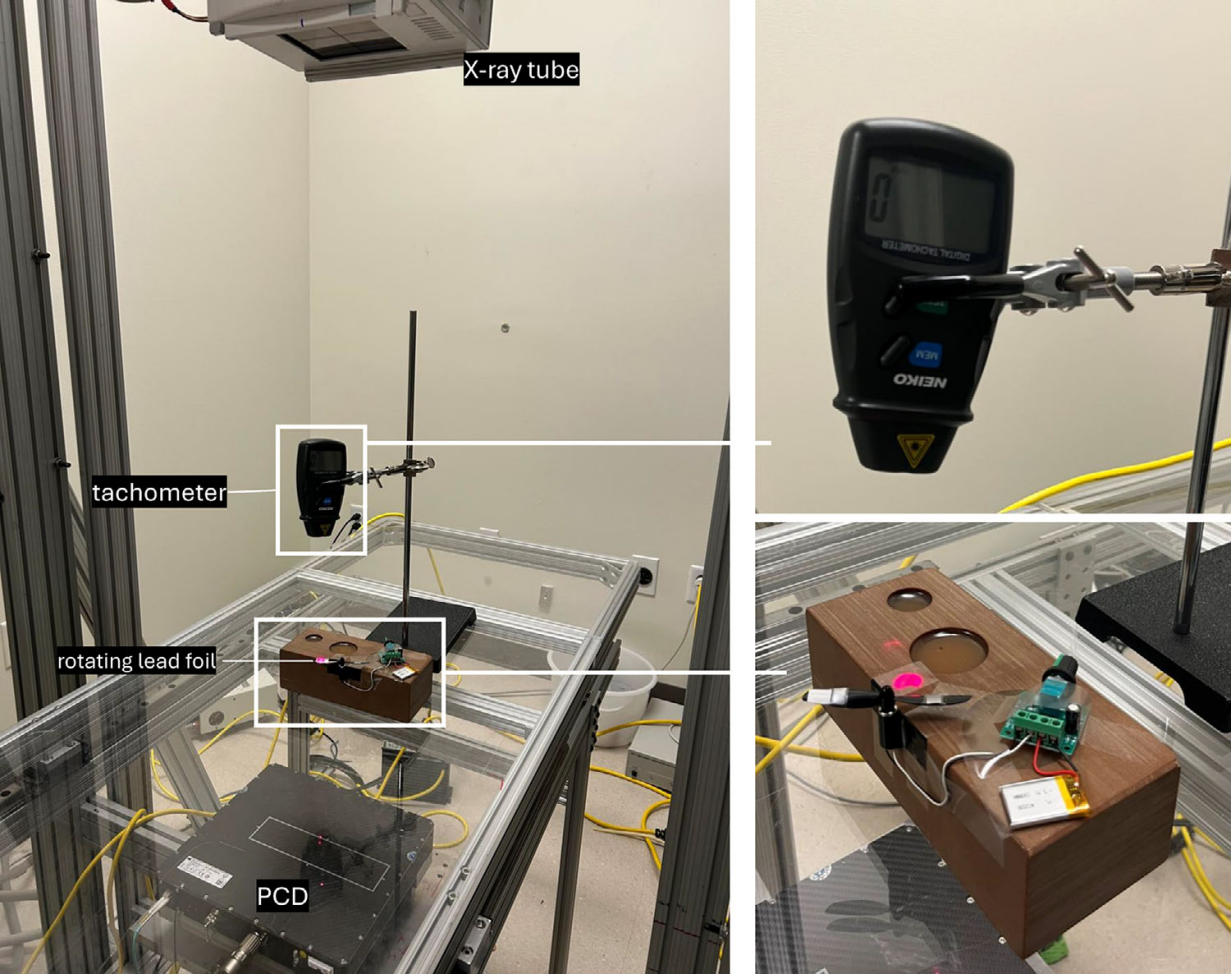
Experimental setup. An x‐ray tube illuminates a rotating fan with affixed lead foils, while a laser tachometer independently measures the angular velocity. A PCD positioned beneath the object records projection images during rotation.

Experiments were conducted using a photon‐counting detector (Model DC‐THOR.10G, Varex Imaging, Salt Lake City, Utah, USA). The detector incorporates a 2.0 mm‐thick CdTe sensor with a native pixel pitch of 100 μm and an active imaging area of 200 × 50 mm^2^. The lowest energy threshold was set to 20 keV to suppress electronic noise. X‐ray illumination was provided by a rotating‐anode x‐ray tube (model G1592 with B‐180H housing, Varex Imaging, Salt Lake City, Utah, USA) driven by an 80 kW high‐frequency high‐voltage generator (Indico 100, CPI Inc., Canada). The tube potential was fixed at 120 kVp. The nominal focal‐spot size was 0.6mm. An overview of the experimental setup is shown in Figure 3.

The source‐to‐detector distance was set to 102 cm. The rotating lead‐foil object was positioned with a source‐to‐object distance of 76 cm. To avoid count saturation at low frame rates, the tube current was set to 50 mA for acquisitions at 300 fps and to 100 mA for other acquisitions with higher PCD frame rates. The x‐ray exposure was not adjusted to maintain a constant number of detected quanta per active acquisition window; thus, the quantum noise level varied across frame‐rate settings. Maintaining a constant number of detected quanta would require increasing the tube current approximately in inverse proportion to the active‐window duration, which was not practical at high frame rates because of potential tube heating and the maximum available tube‐current setting of the system.

A plastic two‐blade fan driven by a DC motor was used as the rotating test object. Two narrow strips of lead foil were affixed to the fan blades to provide sharply defined attenuating edges for motion‐blur analysis while maintaining rotational balance.

The fan speed was continuously adjusted using a pulse‐width‐modulation (PWM) controller to ensure that a measurable motion‐blur width was present at all tested frame rates.

A laser‐reflective foil was attached to one fan blade to enable non‐contact measurement of angular velocity. The rotational speed was measured using a laser tachometer (model 20713A, NEIKO/Ridgerock Tools Inc., Corona, California, USA) with a sampling rate of 1 Hz. The tachometer output was recorded with image acquisition to acquire independent information needed to estimate the angular velocity during each exposure.

The detector was operated in Mode 2 (1×1 binning, 12‐bit digitization) for frame rates between 50 and 500 fps, Mode 5 (1×1 binning, 12‐bit digitization) for frame rates between 500 and 1637.62 fps, and Mode 6 (2×2 binning, 16‐bit digitization) for frame rates exceeding 1637.62 fps. For each detector setting, two sets of projections were acquired: one set without any object in the beam for flat‐field normalization, another set with an object rotating at a constant angular velocity. Each set consisted of 500 frames.

### 3.2 Experimental data

#### 3.2.1 Detector pixel value corrections

All projection frames were first corrected for detector gaps and dead pixels using the correction procedures described below. Isolated dead pixels and small clusters of defective pixels were identified based on statistical standard‐deviation maps computed across projection frames. Because defective pixels and detector gaps are static, they exhibit negligible temporal variance, whereas properly functioning detector pixels show measurable count fluctuations arising from electronic noise. After identification, the defective pixels and gaps were replaced using a bilinear interpolation scheme based on neighboring valid detector pixels. Each acquired projection image was subsequently normalized by an averaged open‐beam air frame to compensate for pixel‐wise sensitivity variations.

#### 3.2.2 Detector timing definitions

For clarity, the timing terminology used in this work is summarized here. A detector frame has a total duration T, referred to as the frame time. Within each frame, photons are actively counted only during a finite interval of duration τ, which is referred to as the active window duration. The remaining portion of the frame time, T−τ, does not contribute to signal formation and is referred to as the inactive window.

According to manufacturer documentation, the inactive window consists of a combination of readout time and internal gap time, during which pixel data are transferred and detector acquisition is inactive. Although the detector remains under continuous x‐ray exposure during these intervals, incident photons are not recorded and therefore do not contribute to image formation.

#### 3.2.3 Center of rotation determination

**FIGURE 4:**
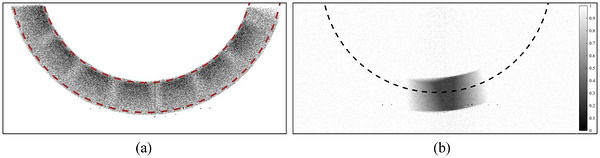
Determination of the rotation center and angular profile extraction.(a) Estimation of the object center of rotation as inferred from projection data. A subset of frames containing the rotating lead‐foil blades was averaged to form a composite image, producing a dark annulus corresponding to the blade trajectory. Circles drawn using the estimated center appear concentric with the annulus, confirming the accuracy of the center location. (b) Illustration of angular profile extraction. The dashed arc indicates the circular path along which the ESF was extracted using the estimated rotation center.

To enable extraction of angular count profiles, the center of rotation of the object was first estimated directly from the projection data. A subset of seven projection frames acquired with 1×1 detector binning and sixteen frames acquired with 2×2 binning were used for this estimation. Because the rotating lead‐foil blades remained within the field of view across these frames, temporal averaging produced a composite image exhibiting a dark annular feature corresponding to the blade trajectory. The center of rotation was empirically determined to allow two conformal arcs to be drawn that enclose the blurred image, as illustrated in Figure 4a. This procedure established a consistent angular coordinate system for subsequent extraction of count profiles along circular arcs.

#### 3.2.4 Angular profile and ESF extraction

Using the estimated center of rotation, angular count profiles were extracted along circular arcs intersecting the blurred lead–foil edge (Figure 4b). The arc radius was selected such that the arc reliably traversed the blurred edge region, as illustrated in Figure 4b. In the example shown, the arc radius was r=28mm. Detector pixel values along each arc were sampled and mapped to angular coordinates to form an edge spread function (ESF).

To ensure consistent angular sampling, the measured detector counts were resampled onto a uniformly spaced angular grid using bilinear interpolation. ESFs were extracted independently for each acquired frame.

#### 3.2.5 Active window duration estimation

**FIGURE 5:**
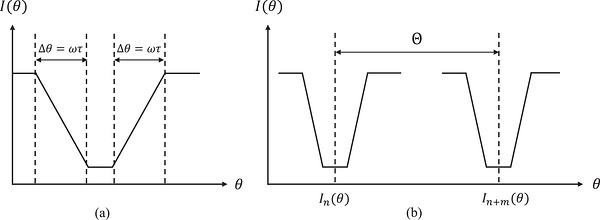
Illustration of rotational motion blur and angular displacement measurement. (a) Angular count profile I(θ) of a blurred lead‐foil edge. The transition width Δθ=ωτ corresponds to the angular distance swept by the object during the active window. (b) Angular displacement Θ between recurring features in frames n and n+m, which is used to estimate the observed frame rate via $\pm \Theta +2k\pi =m\omega /f_{\mathrm{obs}}$.

For each nominal frame‐rate setting ($f_{\mathrm{nom}}$), angular profiles were extracted from individual image frames to form ESFs. As derived in the theoretical analysis, motion blur due to finite active window duration produces an inverted trapezoidal ESF when the object angular width exceeds the blur width (Figure 5).

Accordingly, each trapezoid shape ESF was modeled using a five‐segment piecewise linear function consisting of constant plateaus connected by linear transition ramps:

(18) $$f\left(\theta \right)=\left\{\begin{array}{cc} a_{1}, & \theta <c_{1},\\ a_{1}+\frac{a_{2}-a_{1}}{c_{2}-c_{1}}\left(\theta -c_{1}\right), & c_{1}\leq \theta <c_{2},\\ a_{2}, & c_{2}\leq \theta <c_{3},\\ a_{2}+\frac{a_{3}-a_{2}}{c_{4}-c_{3}}\left(\theta -c_{3}\right), & c_{3}\leq \theta <c_{4},\\ a_{3}, & \theta \geq c_{4}, \end{array}\right.$$

Let $\Delta \theta_{1}=c_{2}-c_{1}$ and $\Delta \theta_{2}=c_{4}-c_{3}$ denote the angular widths of the leading and trailing transition ramps, respectively. The effective detector active window duration τ was estimated for each frame from the average ramp width as

(19) $$\tau =\frac{\Delta \theta_{1}+\Delta \theta_{2}}{2\;\omega },$$

where ω is the independently measured angular velocity of the rotating object. Uncertainty in the active window duration estimate was quantified as the standard deviation of τ across the analyzed frames.

The nonlinear least‐squares optimization function lsqcurvefit in MATLAB (MathWorks, Natick, Massachusetts, USA) was employed to estimate the optimal parameters in Equation (18) by minimizing the sum of squared residuals between the measured data and the model predictions.

#### 3.2.6 Frame rate estimation

The observed detector frame rate, $f_{\mathrm{obs}}$, was estimated from the angular displacement of ESFs measured in the projection images. As illustrated in Figure 5b, the angular displacement Θ between ESFs extracted from frames n and n+m satisfies

(20) $$\pm \Theta +2k\pi =\frac{m\omega }{f_{\mathrm{obs}}},$$

where m denotes the frame separation and k is an integer accounting for possible angular wrap‐around, the ± sign accounts for the dependence of the displacement sign on the chosen angular coordinate system and the rotation direction.

To determine Θ, ESFs were extracted from pairs of projection image frames separated by an empirically selected frame interval m. A fixed normalized intensity threshold of 0.6 was applied to each ESF to isolate the attenuated portion of the profile. For each ESF, the angular coordinates of all samples falling below the threshold were identified, and the median of these coordinates was taken as a robust estimate of the ESF location. The angular displacement Θ was then defined as the difference between the median positions obtained from frames n and n+m.

This median‐based definition mitigates sensitivity to noise and residual ESF asymmetry. The observed frame rate $f_{\mathrm{obs}}$ was obtained by substituting the measured Θ into Equation (20). Measurement uncertainty was estimated from the variation of Θ across multiple frame pairs.

#### 3.2.7 Temporal duty cycle

The temporal duty cycle quantifies the fraction of each frame time during which the detector actively counts photons. For an observed frame rate $f_{\mathrm{obs}}$, the corresponding frame time is $T=1/f_{\mathrm{obs}}$. If the active window duration per frame is τ, the duty cycle is defined as

(21) $$\mathrm{Duty}\;\mathrm{Cycle}=\frac{\tau }{T}=\tau \;f_{\mathrm{obs}}.$$

This definition assumes that detector signal accumulation occurs during a contiguous interval of duration τ within each frame, while the remaining portion of the frame time corresponds to inactive detector operation. The duty cycle therefore provides a metric describing the balance between temporal resolution and photon utilization efficiency in high‐speed PCD readout modes.

## 4 RESULTS

In this section, we present simulation and experimental results that illustrate the temporal characteristics of the detector as they relate to motion‐induced image blur. The results are organized to separately examine the effects of object motion and detector frame rate.

### 4.1 Image blur induced by translational and rotational motion: Simulation studies

Experimental measurements in this work were conducted using rotational motion profiles, owing to their relative simplicity in experimental implementation. To provide complementary physical insight, simulation studies were performed to illustrate image blurring induced by both translational and rotational object motion.

**FIGURE 6:**
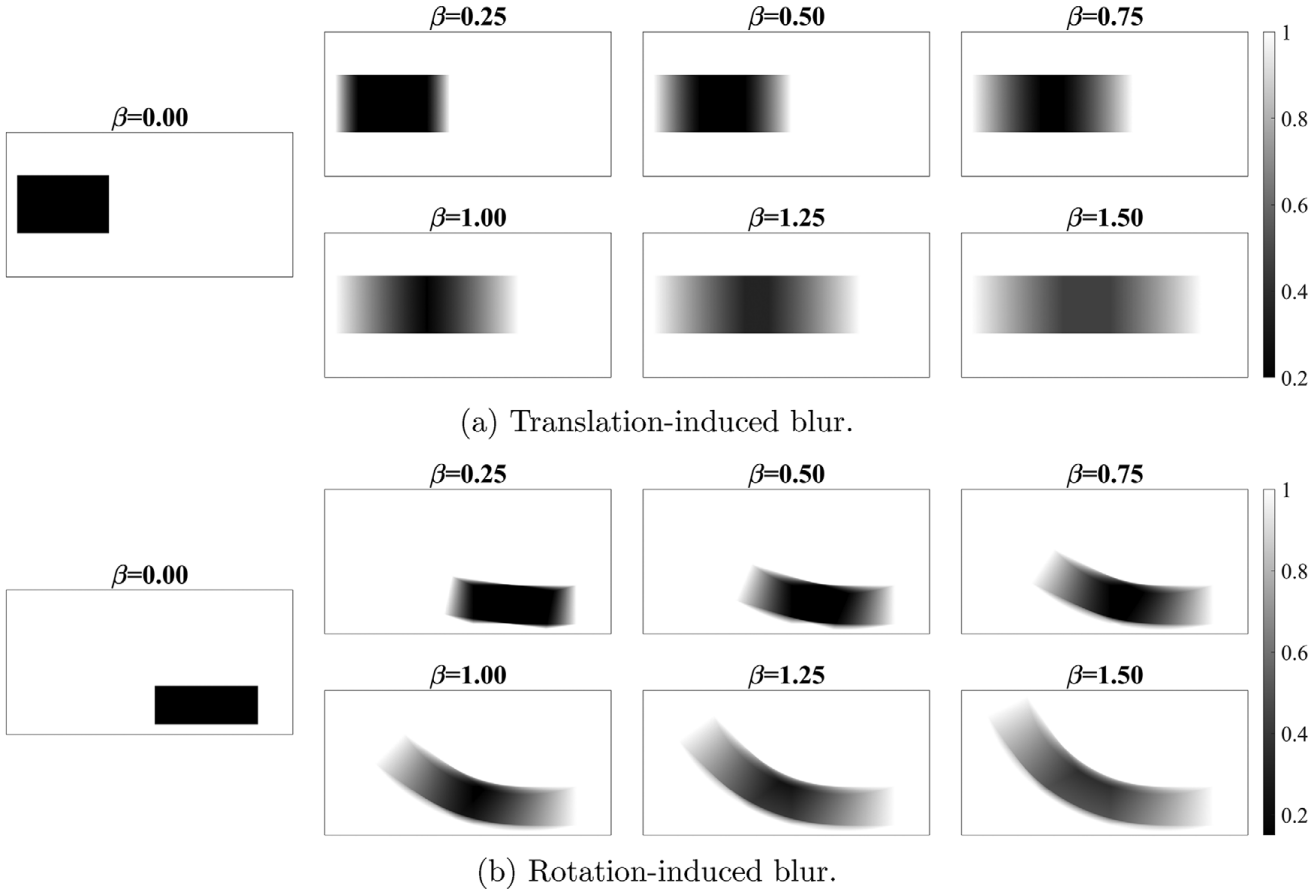
Simulated translational (top) and rotational (bottom) motion blur for increasing values of the normalized blur parameter β. As β increases, the ESF evolves from a trapezoidal profile with a central plateau to a triangular profile at β=1, followed by reduced contrast for β>1, according to the theoretical motion‐blur model.

Simulations were carried out for both the low‐speed (β<1.0) and high‐speed (β≥1.0) regimes. As illustrated in Figure 6, for both translational and rotational motion, increasing the dimensionless characteristic parameter from β=0.25 to β=1.50 leads to progressively increased edge blurring. The qualitative evolution of the blurred edge is consistent across the two motion profiles.

These motion‐blurred images correspond directly to the modeled edge response functions previously presented in Figure 2, which quantify the effects of motion blur through analysis of ESFs extracted from the simulated images.

### 4.2 Edge spread function (ESF)

**FIGURE 7:**
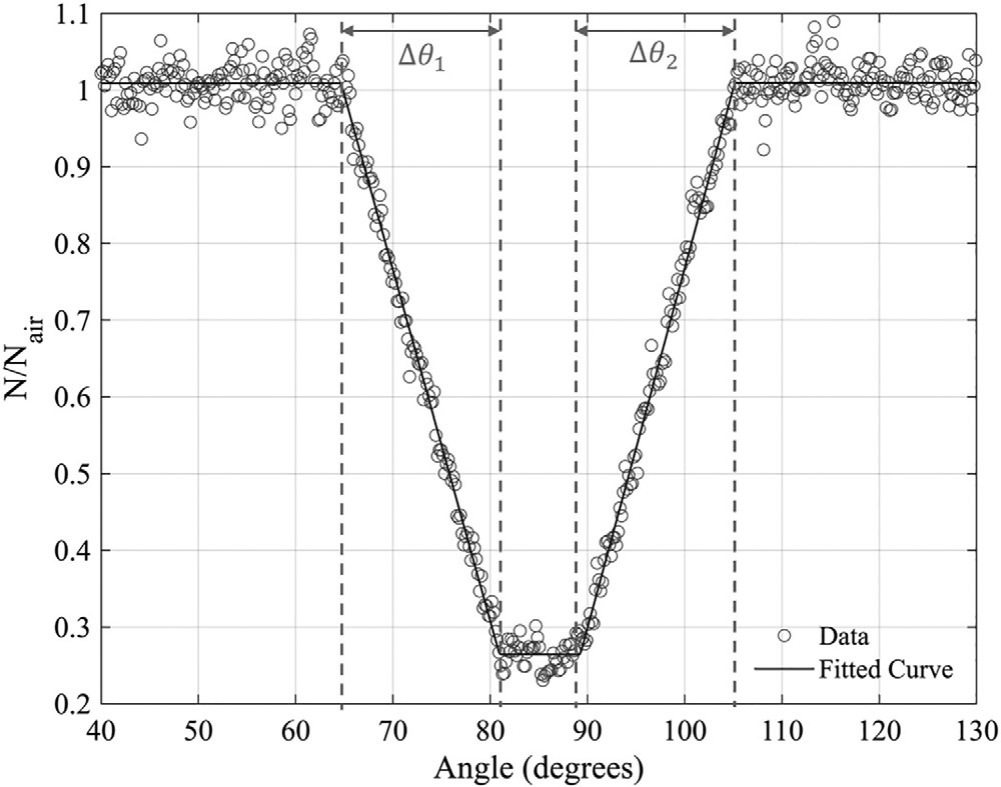
Measured angular ESF data points and the corresponding piecewise linear fit (solid line). Vertical dashed lines indicate the boundaries of the linear transition regions. The angular ramp widths $\Delta \theta_{1}$ and $\Delta \theta_{2}$ are obtained from the fitted model and used to estimate the detector active window duration.

For each selected detector frame rate and object rotation speed, the edge profile was extracted and fitted with the trapezoidal model described above in order to determine the corresponding model parameters. Representative results are shown in Figure 7.

For all detector frame rates and rotation speeds investigated in this work, the corresponding fitted parameters, as defined in Equation (18), are summarized in Table 1. As shown in the table, the baseline image intensity values outside the blurred region, characterized by the parameters $a_{1}$ and $a_{3}$, are essentially identical, as expected for a uniform background. The plateau intensity $a_{2}$ remains nearly constant across all experimental conditions. The angular transition widths $\Delta \theta_{1}$ and $\Delta \theta_{2}$ exhibit the expected symmetry, $|\Delta \theta_{1}|=|\Delta \theta_{2}|$, while their magnitudes increase systematically with increasing motion‐induced blur.

**TABLE 1:** Fitted parameters and fan angular speed (mean ± std) across 15 frame rates.

$f_{\mathrm{nom}}$	ω (rad/s)	$a_{1}$	$a_{2}$	$a_{3}$	$\Delta \theta_{1}\;{(}^{\circ })$	$\Delta \theta_{2}\;{(}^{\circ })$
300	94.9±0.5	1.01±0.00	0.25±0.00	1.01±0.01	17.4±0.1	17.7±0.2
600	150±1	1.00±0.00	0.27±0.00	1.00±0.00	13.1±0.2	13.2±0.1
900	321±2	1.01±0.00	0.27±0.00	1.01±0.00	17.7±0.2	17.8±0.2
1200	362±1	1.01±0.00	0.27±0.00	1.01±0.00	14.3±0.2	14.5±0.3
1500	545±4	1.01±0.00	0.27±0.00	1.01±0.00	16.2±0.2	16.6±0.2
1800	606±4	1.01±0.00	0.27±0.00	1.01±0.00	14.0±0.2	14.1±0.2
2100	641±5	1.02±0.00	0.27±0.00	1.02±0.00	11.9±0.2	12.0±0.1
2400	709±7	1.02±0.00	0.27±0.00	1.02±0.00	11.0±0.2	11.0±0.2
2700	684±7	1.02±0.00	0.28±0.00	1.02±0.00	8.8±0.2	8.9±0.2
3000	709±4	1.02±0.00	0.28±0.00	1.02±0.00	7.5±0.1	7.7±0.2
3300	717±3	1.02±0.00	0.27±0.00	1.02±0.00	6.4±0.1	6.5±0.2
3600	726±2	1.02±0.00	0.27±0.00	1.02±0.00	5.4±0.2	5.6±0.3
3900	749±3	1.04±0.01	0.28±0.00	1.04±0.00	4.8±0.2	4.8±0.3
4200	780±10	1.04±0.01	0.28±0.00	1.03±0.01	4.1±0.2	4.1±0.2
4500	786±3	1.04±0.01	0.28±0.01	1.04±0.01	3.3±0.2	3.6±0.3

### 4.3 Active window duration and observed frame rate

**FIGURE 8:**
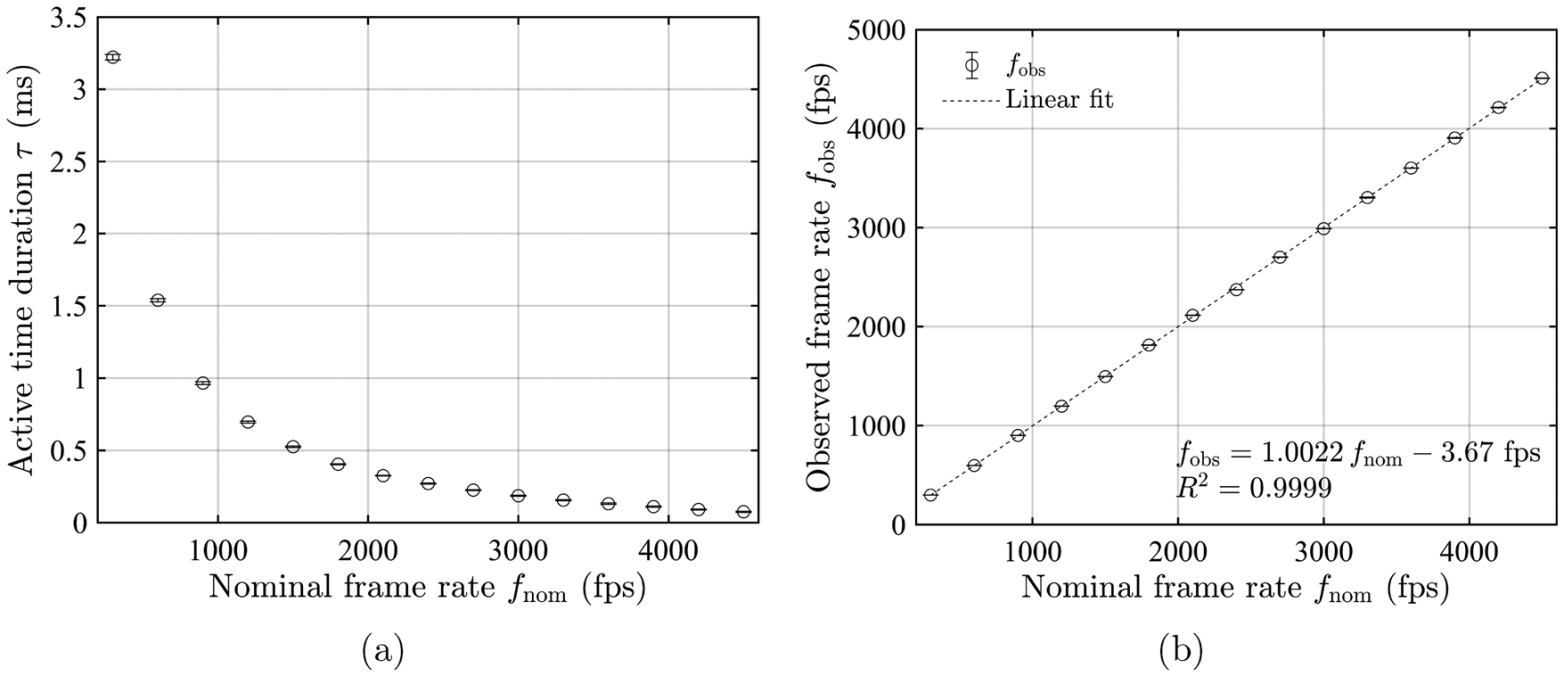
Measured active window duration τ and observed frame rate $f_{\mathrm{obs}}$ measurements. (a) Measured active window duration τ as a function of the nominal frame rate $f_{\mathrm{nom}}$, exhibiting an approximately inverse dependence. (b) Observed frame rate $f_{\mathrm{obs}}$ versus the nominal setting $f_{\mathrm{nom}}$, showing close agreement over the full tested range, with a small offset of approximately 3.7 fps.

Figure 8a shows the measured active window duration τ as a function of the nominal frame rate $f_{\mathrm{nom}}$. The active window duration decreases monotonically with increasing frame rate.

Figure 8b presents the observed frame rate $f_{\mathrm{obs}}$ as a function of the nominal setting $f_{\mathrm{nom}}$. The observed frame rate closely follows the nominal value across the entire range. A linear fit yields

$$f_{\mathrm{obs}}=1.0022\;f_{\mathrm{nom}}-3.67,$$

with a coefficient of determination $R^{2}=0.9999$. The fitted slope is close to unity, and the constant offset is small relative to the absolute frame rate, indicating that the detector timing accurately implements the specified frame‐rate settings.

### 4.4 Inactive window and temporal duty cycle

**FIGURE 9:**
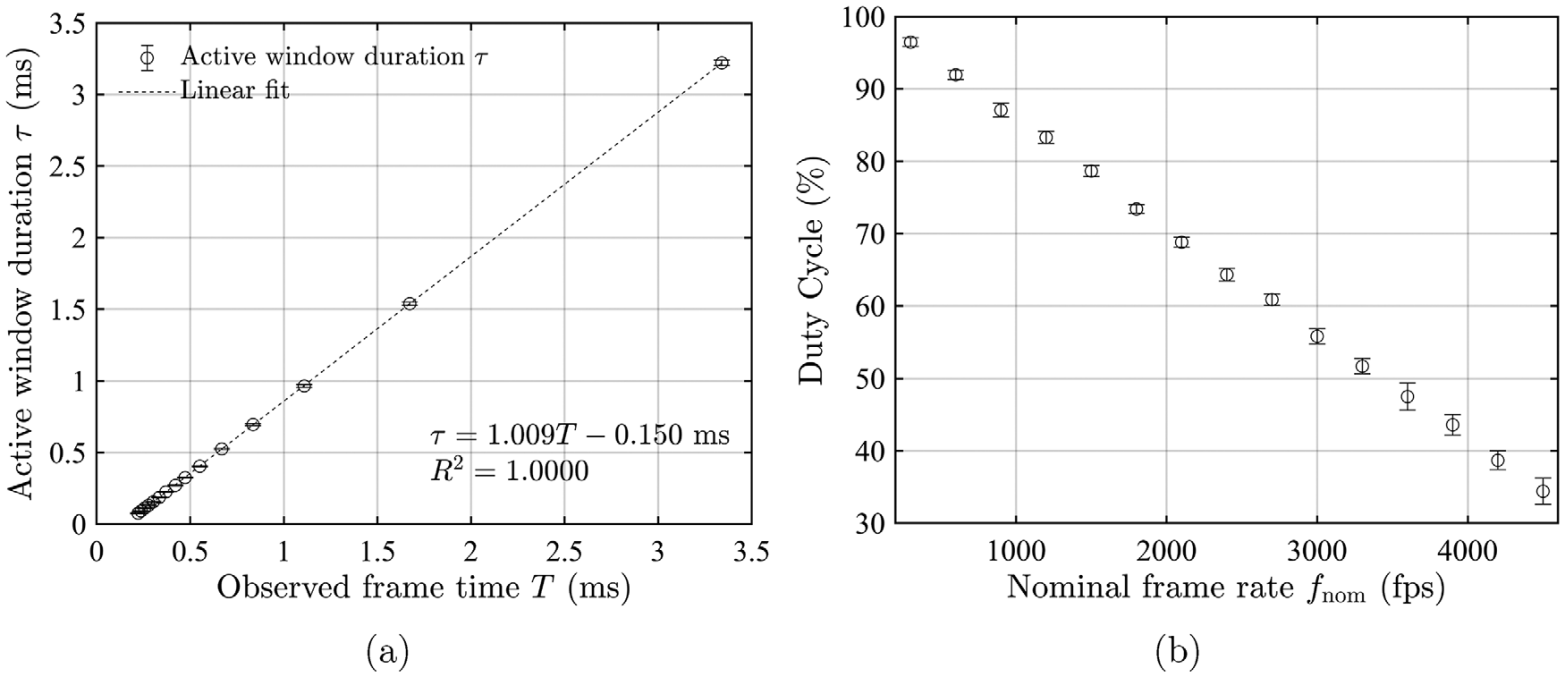
Inactive window and temporal duty cycle. (a) Active window duration τ as a function of the observed frame time $T=1/f_{\mathrm{obs}}$, revealing a linear relationship with a non‐zero intercept, which indicates a constant inactive window per frame. (b) Temporal duty cycle as a function of the nominal frame rate, showing a systematic reduction at higher frame rates due to inactive window.

Using the results shown in Table 1, Equation (19) was used to estimate the active window duration τ. Figure 9a shows the measured active window duration τ plotted as a function of the observed frame time $T=1/f_{\mathrm{obs}}$. A strong linear relationship is observed over the full range of frame times. A linear fit yields

τ=1.009T−0.150ms,

with a coefficient of determination $R^{2}=1.0000$. The fitted slope is close to unity, indicating that the active window duration scales nearly proportionally with the frame time. The non‐zero intercept of approximately 0.15 ms corresponds to a frame‐rate‐independent time interval within each frame during which photon counting does not occur.

Duty cycles at different frame rates were calculated using Equation (21). Figure 9b shows the duty cycle, defined as the ratio of the active window duration to the frame time, as a function of the prescribed frame rate. The results clearly show that the duty cycle decreases systematically with increasing frame rate, dropping from above 90% at low frame rates to below 40% at the highest tested values. This trend reflects the increasing relative contribution of inactive window as the frame time decreases.

## 5 DISCUSSION

This work establishes an imaging physics framework for experimentally characterizing the temporal response of an x‐ray detector using image‐domain motion blur measurements. By combining a rigorous theoretical model of motion‐induced blur formation with controlled object motion independently characterized by tachometer measurements, the study provides a direct link between the measured projection images and the detector temporal integration process. The theoretical framework captures the essential relationship among object kinematics, temporal integration, and image formation, enabling quantitative interpretation of experimentally observed blur features.

A key experimental finding is that the observed frame rate closely follows the nominal detector setting, whereas the active window occupies only a fraction of the frame time. The measured active window duration was consistently shorter than the nominal frame time due to the presence of a frame‐rate‐independent inactive window. This behavior was observed across all tested frame‐rate settings and detector operating modes, indicating that the inactive window is intrinsic to the tested detector readout architecture rather than a configurable parameter. Importantly, this inactive window should be regarded as a practical detector‐performance characteristic of the specific readout architecture and operating mode, rather than as a fundamental physical limitation common to all x‐ray detectors. The existence of this interval provides a physical explanation for the systematic reduction in temporal duty cycle at higher frame rates.

An important implication of the frame‐rate‐independent inactive window identified in the tested detector concerns radiation dose efficiency in practical applications. During the inactive window of each frame, if x‐rays continue to irradiate the object while the detector is not responsive, the radiation exposure delivered during this interval does not contribute to the measured signal. As a result, a portion of the incident radiation does not contribute to the recorded projection data, potentially reducing radiation dose efficiency. This effect could be further quantified in future work using metrics such as detective quantum efficiency (DQE). The magnitude of this dose‐efficiency penalty depends strongly on the frame rate. In many current time‐resolved projection imaging applications in interventional radiology, typical frame rates are well below 30 fps. Under such conditions, the inactive window accounts for less than 0.5% of the frame period, and its impact is expected to be relatively small. Even with this inactive window, the detector may retain value for selected high‐frame‐rate applications, such as flow imaging in interventional radiology, where improved temporal sampling may help visualize detailed flow dynamics.^1^, ^3^, ^4^ In contrast, if the detector were used for CT acquisitions, including diagnostic CT applications in which detector frame rates on the order of several thousand frames per second may be required, the frame period would be compressed to the sub‐millisecond range. In this regime, a frame‐rate‐independent inactive window can become comparable to the acquisition time available for each frame and therefore represents a more significant limitation.

The experimentally measured ESFs exhibit well‐defined linear transition regions and flat plateaus, in close agreement with the piecewise trapezoidal profiles predicted by the theoretical model. This agreement in shape, rather than solely in extracted scalar parameters, provides strong evidence that photon counting occurs predominantly within a single, contiguous temporal window during each frame. The observed ESF morphology thus validates the assumption underlying the theoretical analysis and supports the definition of temporal duty cycle as the ratio between effective active window duration and frame time. However, non‐temporal blur sources, including finite focal‐spot size and detector aperture, may introduce systematic uncertainty and should be quantified to place the measured temporal response in proper context. As shown in the Results section, the smallest measured angular transition width in this study was $\Delta \theta_{\min}=3.3^{\circ }$. The detector‐aperture contribution was estimated as $\Delta \theta_{\mathrm{pix}}\approx p/r$, where p=0.1mm is the detector pixel size and r=28mm is the ESF extraction radius, giving $\Delta \theta_{\mathrm{pix}}\approx 0.2^{\circ }$, or about 6% of $\Delta \theta_{\min}$. The finite focal‐spot blur was estimated as $b_{f}=f_{\mathrm{spot}}\left(\mathrm{SDD}-\mathrm{SOD}\right)/\mathrm{SOD}$, with $f_{\mathrm{spot}}=0.6\;\mathrm{mm}$, SDD=102cm, and SOD=76cm. This gives $b_{f}\approx 0.21\;\mathrm{mm}$, corresponding to approximately $0.42^{\circ }$, or about 13% of $\Delta \theta_{\min}$. Therefore, both geometric blur contributions were substantially smaller than the measured ESF broadening. In addition, the detector manual reports 0% lag under the relevant operating condition, suggesting that detector lag is unlikely to contribute appreciably. These estimates support the interpretation that the fitted ESF transition widths were dominated by temporal motion blur rather than by detector aperture, focal‐spot blur, or detector lag.

The results further clarify the distinction between nominal frame rate and effective temporal resolution. Although increasing the frame rate reduces the frame time, the active window duration does not scale proportionally due to the presence of the inactive window. Consequently, frame rate alone is insufficient to characterize temporal fidelity in high‐speed imaging. Instead, active window duration governs the degree of motion blur and must be considered explicitly when evaluating temporal performance and dose efficiency, particularly in applications such as computed tomography where high frame rates are routinely employed.

The use of rotational motion in this study provides an experimentally robust means of probing detector temporal behavior. Expressing motion blur in angular coordinates yields a measurement that is invariant to geometric magnification, thereby reducing sensitivity to object–detector distance and alignment uncertainties. This geometric invariance, combined with independent tachometer measurements of angular velocity, enables a reliable extraction of the active window duration directly from projection images under realistic imaging conditions.

Finally, the limitations of the proposed motion‐blur–based approach must be acknowledged.

First, in our experiments, the tube current was set to 50mA for the 300fps acquisition and to 100mA for acquisitions at higher PCD frame rates, because further increases would have exceeded the operating capacity of the angiography x‐ray tube used in our system. However, to assess the resulting experimental variability, repeated measurements were performed under each acquisition condition, and the empirical variations were reported in Table 1.

Second, reliable extraction of detector active window duration requires that the object motion be chosen such that the characteristic blur features remain robustly observable in the image domain. As established by the theoretical model, when the blur length approaches the object width (β=Δx/a→1), the fully shadowed plateau in the normalized contrast profile collapses to a single point. For β>1, the maximum contrast falls below unity because no detector position remains continuously shadowed throughout the active window, necessitating inference of τ from contrast amplitude and plateau width rather than from transition width.

Third, in the case of rotational motion, an additional constraint arises from the periodicity of angular motion. The rotational formulation assumes a single‐pass regime in which the angular blur width Δθ=ωτ remains below the first wrap‐around threshold, such that Δθ<2π and each angular location is shadowed at most once during a single active window. When this condition is violated, multiple shadowing events occur within the same active window, and the measured signal increasingly reflects a rotational time average rather than a single‐pass motion‐blur profile. Under these circumstances, the trapezoidal contrast model underlying the present method no longer applies, and reliable extraction of τ from blur geometry becomes impractical.

In the present study, the object rotation speed was deliberately selected to satisfy both β<1 and Δθ≪2π, ensuring that plateau and ramp features were well defined and that parameter extraction was most robust. Consequently, the experimental results do not probe the β>1 regime nor the onset of angular wrap‐around. Future studies will extend the measurements to β>1 while remaining below the first wrap‐around threshold, in order to experimentally validate the model predictions in the sub‐unity contrast regime and to assess the robustness of active window duration estimation under these conditions.

## 6 CONCLUSION

This study developed and experimentally validated a motion‐blur‐based approach for quantifying the frame‐level temporal response of high‐frame‐rate x‐ray detectors at the projection‐image level. Validation was performed using a high‐frame‐rate photon‐counting detector. A rigorous theoretical framework was established to relate object motion, detector active window duration, and image‐domain blur formation, enabling direct and physically interpretable extraction of the active window duration from measured projection data using independently characterized motion.

Experimental results demonstrate that, although the observed frame rate closely matches the nominal detector setting, the active window duration occupies only a fraction of the frame time due to the presence of a frame‐rate‐independent inactive window. As a consequence, the detector temporal duty cycle decreases systematically with increasing frame rate, with direct implications for temporal fidelity and radiation dose utilization. The measured edge‐spread functions exhibit trapezoidal profiles in close agreement with theoretical predictions, providing strong evidence that photon counting occurs predominantly within a single, contiguous temporal window during each frame.

Overall, the results demonstrate that nominal frame rate alone is insufficient to characterize detector temporal performance. Instead, the effective active window duration and duty cycle must be considered explicitly, particularly at high frame rates where reduced active window duration substantially lowers dose efficiency. Motion‐blur analysis therefore provides a robust and physics‐grounded tool for assessing the temporal behavior of x‐ray detectors under realistic imaging conditions.

## FUNDING INFORMATION

This work is partially supported by NIH Grant R01EB032474. One of the authors, G.H.C., received partial support from the WARF Named Professorship, a chaired professorship bestowed by the University of Wisconsin‐Madison's Office of the Vice Chancellor for Research and Graduate Education, with funding from the Wisconsin Alumni Research Foundation (WARF).

## CONFLICT OF INTEREST STATEMENT

The authors declare no conflicts of interest.

## APPENDIX A

In this Appendix, we provide the detailed derivation of Equation (14). Let the object support be the interval $\left[-\frac{a}{2},\;\frac{a}{2}\right]$. For a detector position x, the function g(x)=x−Δx=x−vτ defines a one‐parameter family of straight lines with slope 1 and intercept −Δx in the (x,g) plane. The overlap fraction η(x) is given by the vertical extent of the parallelogram bounded by the two lines g(x)=x−Δx and g(x)=x and the two horizontal lines $g\left(x\right)=\pm \frac{a}{2}$, normalized by Δx.

When the object width satisfies a>Δx, the detector active window can be fully contained within the object support over a nonempty range of detector positions. A straightforward case analysis yields the trapezoidal profile:

(A1)
$$\begin{array}{c} \eta \left(x\right)=\left\{\begin{array}{cc} 0, & x\leq -a/2,\\ \frac{x+a/2}{\Delta x}, & -a/2<x<-a/2+\Delta x,\\ 1, & -a/2+\Delta x\leq x\leq a/2,\\ \frac{a/2+\Delta x-x}{\Delta x}, & a/2<x<a/2+\Delta x,\\ 0, & x\geq a/2+\Delta x. \end{array}\right. \end{array}$$

Equation (A1) describes a trapezoidal contrast profile with a central plateau of width a−Δx and two linear transition regions, each of width Δx. The ramp width therefore corresponds directly to the distance traveled by the object during the active window.

When the object width satisfies a≤Δx, no detector position can remain fully shadowed throughout the active window. In this regime, the overlap length is bounded above by the object width a, and the maximum normalized contrast decreases to $\eta_{\max}=a/\Delta x<1$. Explicit evaluation of the overlap length, L(x), yields

(A2)
$$\eta \left(x\right)=\left\{\begin{array}{cc} 0, & x\leq -a/2,\\ \frac{x+a/2}{\Delta x}, & -a/2<x<a/2,\\ \frac{a}{\Delta x}, & a/2\leq x\leq -a/2+\Delta x,\\ \frac{a/2+\Delta x-x}{\Delta x}, & -a/2+\Delta x<x<a/2+\Delta x,\\ 0, & x\geq a/2+\Delta x. \end{array}\right.$$

In contrast to Equation (A1), the profile exhibits a reduced plateau height of a/Δx and a plateau width of Δx−a, while the linear transition regions each have width a. The limiting case a=Δx corresponds to a triangular profile in which the plateau collapses to a single point, and the two expressions join continuously.

The two regimes discussed above can be expressed within a single, unified formulation by introducing the auxiliary quantities

(A3)
$$w\equiv \min\left(a,\Delta x\right),\;\eta_{\max}\equiv \min\;\left(1,\frac{a}{\Delta x}\right),$$

which respectively represent the effective transition width and the maximum achievable normalized contrast.

With these definitions, the normalized contrast profile can be written in a regime‐agnostic piecewise form as

(A4)
$$\eta \left(x\right)=\left\{\begin{array}{cc} 0, & x\leq -a/2,\\ \frac{x+a/2}{\Delta x}, & -a/2<x<-a/2+w,\\ \eta_{\max}, & -a/2+w\leq x\leq a/2+\Delta x-w,\\ \frac{a/2+\Delta x-x}{\Delta x}, & a/2+\Delta x-w<x<a/2+\Delta x,\\ 0, & x\geq a/2+\Delta x. \end{array}\right.$$

This is the result presented in Equation (14). As a consistency check, Equation (14) reduces to Equation (A1) in the regime with Δx≤a and to Equation (A2) in the regime with Δx>a. Equation (14) therefore provides a single, self‐consistent description that correctly captures the contrast behavior across both regimes.
